# Pseudolaric Acid B Induces Caspase-Dependent and Caspase-Independent Apoptosis in U87 Glioblastoma Cells

**DOI:** 10.1155/2012/957568

**Published:** 2012-06-20

**Authors:** Muhammad Khan, Bin Zheng, Fei Yi, Azhar Rasul, Zhuyi Gu, Ting Li, Hongwen Gao, Javed Iqbal Qazi, Hong Yang, Tonghui Ma

**Affiliations:** ^1^Central Research Laboratory, Jilin University Bethune Second Hospital, Changchun 130041, China; ^2^College of Life Sciences, Liaoning Normal University, Dalian 116029, China; ^3^Department of Zoology, University of the Punjab, Quaid-e-Azam Campus, Lahore 54590, Pakistan

## Abstract

Pseudolaric acid B (PLAB) is one of the major bioactive components of *Pseudolarix kaempferi*. It has been reported to exhibit inhibitory effect on cell proliferation in several types of cancer cells. However, there is no report elucidating its effect on glioma cells and organ toxicity *in vivo*. In the present study, we found that PLAB inhibited growth of U87 glioblastoma cells in a dose-dependent manner with IC_50_ *~*10 **μ**M. Flow cytometry analysis showed that apoptotic cell death mediated by PLAB was accompanied with cell cycle arrest at G2/M phase. Using Western blot, we found that PLAB induced G2/M phase arrest by inhibiting tubulin polymerization in U87 cells. Apoptotic cell death was only partially inhibited by pancaspase inhibitor, z-VAD-fmk, which suggested that PLAB-induced apoptosis in U87 cells is partially caspase-independent. Further mechanistic study demonstrated that PLAB induced caspase-dependent apoptosis via upregulation of p53, increased level of proapoptotic protein Bax, decreased level of antiapoptotic protein Bcl-2, release of cytochrome c from mitochondria, activation of caspase-3 and proteolytic cleavage of poly (ADP-ribose) polymerase (PARP) and caspase-independent apoptosis through apoptosis inducing factor (AIF). Furthermore, *in vivo* toxicity study demonstrated that PLAB did not induce significant structural and biochemical changes in mouse liver and kidneys at a dose of 25 mg/kg. Therefore, PLAB may become a potential lead compound for future development of antiglioma therapy.

## 1. Introduction

Primary brain tumors are the tumors that originate from various intracranial tissues. More than 60% of brain tumors are gliomas [1]. Glioblastoma multiforme is the most common and lethal primary brain tumor in adults and accounts for at least 80% of malignant gliomas. It is also called grade IV astrocytoma [2–5]. Over 12,000 patients die because of primary brain tumor in United States every year. Despite recent advances in surgery, radiation therapy, and chemotherapy, the median survival rate remains less than one year after diagnosis [1, 6, 7].

Pseudolaric acid B (PLAB) is one of the major diterpenoid compounds isolated from root and trunk bark of *Pseudolarix* *kaempferi* and possesses multiple biological and pharmacological activities including antifungal, antimicrobial, antifertility, and antiangiogenic properties [8, 9]. To date, several pharmacological reports have shown that PLAB induces growth inhibition, cell cycle arrest, and apoptosis in a number of cancer cell lines including breast cancer, colon cancer, hepatocellular carcinoma, melanoma cells [9], liver cancer, cervical cancer, gastric cancer, lung cancer, and leukemia [10, 11]. Further studies have shown that PLAB induces apoptosis via activation of c-Jun N-terminal kinase and caspase-3 in HeLa cells [12], through p53 upregulation in gastric carcinoma MGC803 cells [11], through Bcl-2 downregulation and caspase-3 activation in AGS gastric cancer cells [13], through p53 and Bax/Bcl-2 pathways in human melanoma A375-S2 cells [12] and through activation of JNK and inactivation of ERK in breast cancer MCF-7 cells [9]. In addition, PLAB has induced G2/M phase arrest by activation of the ATM signalling pathway in human melanoma SK-28 cells [9], through p53 and p21 upregulation in breast cancer MCF cells [8] and by inhibiting tubulin polymerization in human microvascular endothelial cells, human leukemia HL-60 cells, Hela cells, and human umbilical vascular endothelial cells [10, 14, 15]. So far, the effect of PLAB on gliomas has not been reported. Furthermore, there is no report on toxicological effects of PLAB on normal cells *in vivo*.

The present study was aimed to examine the growth inhibitory effect of PLAB on U87 glioblastoma cells and toxicological effect of PLAB on normal cells in animal mouse model. The molecular mechanism of PLAB-induced growth inhibition of U87 glioblastoma cells was studied using Western blots. The toxicological effect (hepatotoxicity and renal toxicity) of PLAB was studied in Kunming mice. A histopathology assessment of the liver and kidneys was carried out and correlated with the plasma levels of liver function biomarkers; aspartate aminotransferase (AST), alanine aminotransferase (ALT), total bilirubin (TBIL), and renal function biomarkers; blood urea nitrogen (BUN) creatinine (Cr), respectively.

## 2. Materials and Methods

### 2.1. Chemicals

**Figure 1:**
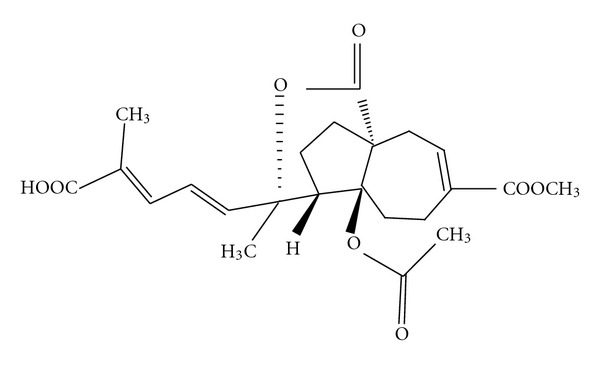
Chemical structure of pseudolaric acid B (PLAB).

Pseudolaric acid B (PLAB) was purchased from Tauto biotech Co., Ltd. (Shanghai, China) and purity (>98%) was determined by HPLC. The chemical structure of PLAB is shown in Figure 1. RNase A, propidium iodide (PI) calcein acetoxymethyl ester (Calcein AM), Hoechst 33258, Dimethyl Sulfoxide (DMSO), [3-(4,5-Dimethylthiazol-2-yl)-2,5-Diphenyltetrazolium Bromide] (MTT), Dulbecco's Modified Eagle's Medium (DMEM), and fatal bovine serum (FBS) were purchased from Sigma (Beijing, China). Apoptosis assay kit, general caspase inhibitor (z-VAD-fmk), p53 inhibitor (PFT*α*), antibodies specific to p53, Bax, Bcl-2, Cytochrome c, Caspase-3, and poly(ADP-ribose) polymerase (PARP) and Tubulin were purchased from Beyotime institute of Technology (Shanghai, China), whereas antibodies specific to cyclin B1 and Cdc2 were purchased from Cell Signalling (China). Antibodies specific to apoptosis inducing factor (AIF), *β*-actin and horseradish peroxidase-conjugated secondary antibodies (goat-anti-rabbit, goat-anti-mouse, rabbit-anti-goat) were purchased from Santa Cruz (Beijing, China).

### 2.2. Cell Culture and Treatments

U87 glioblastoma cells were obtained from American Type Culture Collection (ATCC, USA) and maintained in Dulbecco's Modified Eagle's Medium (DMEM) supplemented with 10% fatal bovine serum (FBS) in 5% CO_2_ at 37°C. Cells were treated with various concentrations of PLAB dissolved in DMSO with a final DMSO concentration of 1% or with DMSO alone for 24 h. DMSO-treated cells were used as control.

### 2.3. Determination of Cell Viability

Cell viability was assessed by MTT assay and live/dead assay as described by us previously [16]. Briefly U87 cells were treated with various concentrations of PLAB (1 to 100 *μ*M) or Doxorubicin (0.25 to 20 *μ*M) for 24 h. Following treatment, the MTT reagent was added (500 *μ*g/mL) and cells were further incubated at 37°C for 4 h. Subsequently 150 *μ*L DMSO was added to dissolve farmazan crystals and absorbance was measured at 570 nm in a microplate reader (Thermo Scientific). Results were expressed as the percentage of MTT reduction, assuming that the absorbance of control cells was 100%. 

Furthermore, live and dead cells were quantified using the fluorescent probes calcein AM and PI. Calcein AM is cell membrane permeable and stains only viable cells, whereas PI is cell membrane impermeable and stains only dead cells. After treatment, cells were collected, washed with phosphate buffered saline (PBS) and incubated with PBS solution containing 2 *μ*M calcein AM and 4 *μ*M PI in the dark for 20 min at room temperature. After washing, cells were resuspended in PBS and analyzed for the fluorescence of calcein and PI by flow cytometry (Beckman Coulter, Epics XL).

### 2.4. DNA Fragmentation by Hoechst 33258 Staining

After treatment with 5 and 10 *μ*M PLAB for 24 h, U87 cells were collected by centrifugation at 1500 rpm for 5 min, washed twice with PBS and fixed with 4% paraformaldehyde at room temperature for 30 min. After centrifugation, cells were washed with PBS, stained with Hoechst 33258 (50 *μ*g/mL) and incubated at 37°C for 30 min. At the end of incubation, cells were washed and resuspended in PBS for the observation of nuclear morphology under fluorescence microscope (Olympus 1 × 71). At the end, 100 nuclei from control and PLAB-treated groups were counted microscopically for the percentage of cleaved nuclei. 

### 2.5. Flow Cytometry Analysis of Apoptosis

U87 cells were treated with 5 and 10 *μ*M PLAB in the presence or absence of z-VAD-fmk and PFT*α* for 24 h. After treatment, both adherent and floating cells were collected, washed with PBS, and resuspended in 200 *μ*L of binding buffer containing 5 *μ*L Annexin V and put in the dark for 10 min according to the kit instructions (Beyotime, Shanghai, China). After incubation, cells were labeled with 10 *μ*L PI and samples were immediately analyzed by flow cytometry (Beckman Coulter, Epics XL).

### 2.6. Flow Cytometry Analysis of Cell Cycle

U87 cells were treated with 5 and 10 *μ*M PLAB for 24 h. Following treatment, cells were harvested, washed with PBS, and fixed with 70% ethanol at 4°C for overnight. After washing twice with PBS, cells were stained with a solution containing 50 *μ*g/mL PI and 100 *μ*g/mL RNase A for 30 min in the dark, at room temperature. The DNA contents were analyzed by flow cytometry (Beckman Coulter, Epics XL).

### 2.7. Protein Extraction and Western Blotting

After drug treatment, adherent and floating cells were collected and proteins were isolated as described previously [16]. Nuclear and cytosolic proteins were extracted using cytosolic and nuclear extraction kit (Keygen, China) according to the manufacturer's instructions. 40 *μ*g proteins were electrophoresed on 10% SDS-PAGE and transferred to PVDF membrane. After blocking with 5% (w/v) nonfat milk and washing with Tris-buffered saline-Tween solution (TBST), membranes were incubated overnight at 4°C with p53 (1 : 1000), BCL-2 (1 : 1000), Bax (1 : 300), Cytochrome c (1 : 200), Caspase-3 (1 : 500), Tubulin (1 : 500), Cyclin B1 (1 : 300), Cdc2 (1 : 300), *β*-actin (1 : 400), and AIF (1 : 1000) antibodies, respectively. After washing, the blots were incubated with horseradish peroxidase-conjugated goat anti-rabbit IgG or goat anti-mouse IgG or rabbit anti-goat IgG secondary antibodies (1 : 1000, Santa Cruz) for 1 h at room temperature. After washing with TBST, signals were detected using ECL plus chemiluminescence kit on X-ray film (Millipore Corporation, Billerica, USA).

### 2.8. Extraction of Polymeric Tubulin

After treating the cells with 10 *μ*M PLAB and 3 *μ*M colchicine, cells were harvested and washed with PBS and polymeric tubulins were extracted as described previously [17]. Briefly the cell pellet was resuspended in 0.4 mL monomeric extraction buffer (20 mM Tris-HCl, pH 6.8, 0.14 M NaCl, 1 mM MgCl2, 1 mM EGTA, 0.5% Nonidet P-40 (NP-40), 0.5 mM PMSF, and 4 *μ*g/mL paclitaxel), centrifuge at 12,000 ×g for 10 min and supernatant was removed. The pellets containing polymeric tubulin were resuspended in WIP cell lysis reagent (BIOSS, Beijing Biosynthesis Biotechnology Co., Ltd.) for 30 min and the supernatants (polymeric tubulins) were collected by centrifugation at 12,000 ×g for 10 min. The polymeric tubulins were subjected to Western blot analysis.

### 2.9. *In Vivo* Studies

*In vivo* studies were conducted on 12–14 week old Kunming mice weighing 43–45 g. The mice were maintained in a specific pathogen-free grade animal facility on a 12 h light/dark cycles at 25 ± 2°C. Mouse procedures were approved by the Experimental Animal Committee of Jilin University. Mice were divided into two groups. Group A (*n* = 5) administered with 50 *μ*L DMSO intraperitoneally; Group B (*n* = 5) administered with PLAB (25 mg/kg body weight) in 50 *μ*L DMSO intraperitoneally. The experiment was conducted over a period of two weeks. DMSO or drug was administered daily for 14 days, once a day. At the first and last day of the experiment, the body weight of each mouse was measured. At the end of experiment, mice were anesthetized using Pentobarbital sodium (50 mg/kg ip), blood was collected via cardiac puncture, allowed to clot for 10 min, centrifuge at 1000 ×g for 10 min at room temperature. Serum was separated and stored at −20°C until analysis. The liver and kidneys were excised and processed for hematoxylin and eosin staining followed standard procedures.

### 2.10. Serum Biomarker Analysis

The toxicological effect of PLAB on liver function was assessed by measuring the serum levels of AST, ALT and TBIL. Nephrotoxicity was determined by measuring the serum levels of BUN and Cr. These biochemical parameters were determined by an automated biochemical analyzer (Hitechi 7170, Japan).

## 3. Statistical Analysis

The results are expressed as Mean ± SEM and statistically compared with control group or within the groups using one way ANOVA followed by Tukey's Multiple Comparison Test. Student's *t*-test was used to determine significance when only two groups were compared and *P* < 0.05 was considered statistically significant.

## 4. Results

### 4.1. PLAB Reduces Cell Viability and Induces Cell Death in U87 Glioblastoma Cells

**Figure 2:**
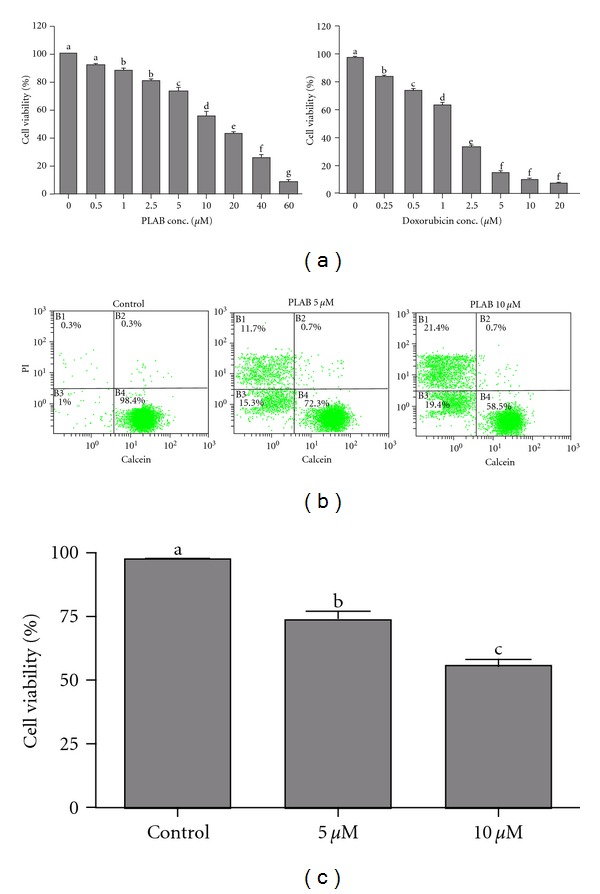
Effect of PLAB on U87 glioblastoma cell viability. (a) U87 cells were treated with indicated concentrations of PLAB and doxorubicin (positive control) for 24 h and cell viability was determined by MTT assay. Data are expressed as Mean ± SEM (*n* = 3). Columns not sharing the same superscript letter differ significantly (*P* < 0.05). (b) U87 cells were treated with DMSO or 5 and 10 *μ*M PLAB for 24 h. Following treatment, cells were harvested, washed with PBS, and stained with 2 *μ*M calcein AM and 4 *μ*M PI in the dark for 20 min. After washing, cells were resuspended in PBS and samples were analyzed by flow cytometry for the quantification of live and dead cells. (c) Data of figure (b) are expressed as Mean ± SEM (*n* = 3). Columns not sharing the same superscript letter differ significantly (*P* < 0.05).

Cell viability was determined by MTT assay. Treatment with PLAB for 24 h inhibited growth of U87 glioblastoma cells in a dose-dependent manner (Figure 2(a)). The inhibition rate was above 85% at 100 *μ*M and the concentration to achieve IC_50_ was 10 *μ*M. A reference drug (Doxorubicin) was used as positive control whose IC_50_ against U87 glioblastoma cells was 1.8 *μ*M (Figure 2(a)). 5 and 10 *μ*M concentrations were selected for further studies. These results were further verified by live/dead assay using flow cytometry. The cells stained and retained calcein are alive and scattered in region B4. The regions B3 and B1 showed dead cells. As shown in Figures 2(b) and 2(c), the viability of U87 glioblastoma cells treated with 5 and 10 *μ*M PLAB for 24 h was significantly lower (74.7 ± 1.83 and 56.87 ± 3.47 versus 97.77 ± 1.45 in control group, *P* < 0.05).

### 4.2. PLAB Induces Apoptotic Cell Death in U87 Glioblastoma Cells

**Figure 3:**
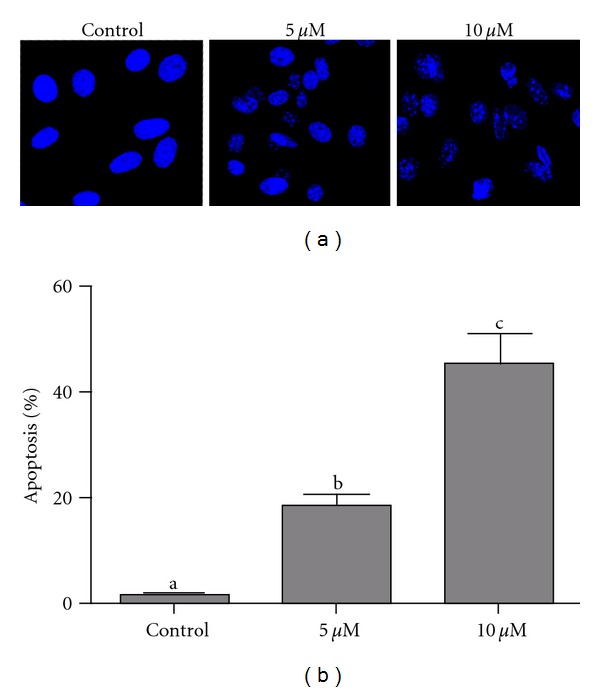
Nuclear morphological changes of U87 glioblastoma cells. (a) U87 cells were treated with DMSO or 5 and 10 *μ*M PLAB for 24 h. After fixing with 4% paraformaldehyde at room temperature for 30 min, cells were stained with Hoechst 33258 (50 *μ*g/mL) in the dark for 30 min. After washing, cells were resuspended in PBS solution and nuclear morphological changes were observed by fluorescence microscope. Finally, 100 nuclei were counted microscopically from each group for the percentage of cleaved nuclei (Apoptosis). (b) Data are expressed as Mean ± SEM (*n* = 3). Columns not sharing the same superscript letter differ significantly (*P* < 0.05).

**Figure 4:**
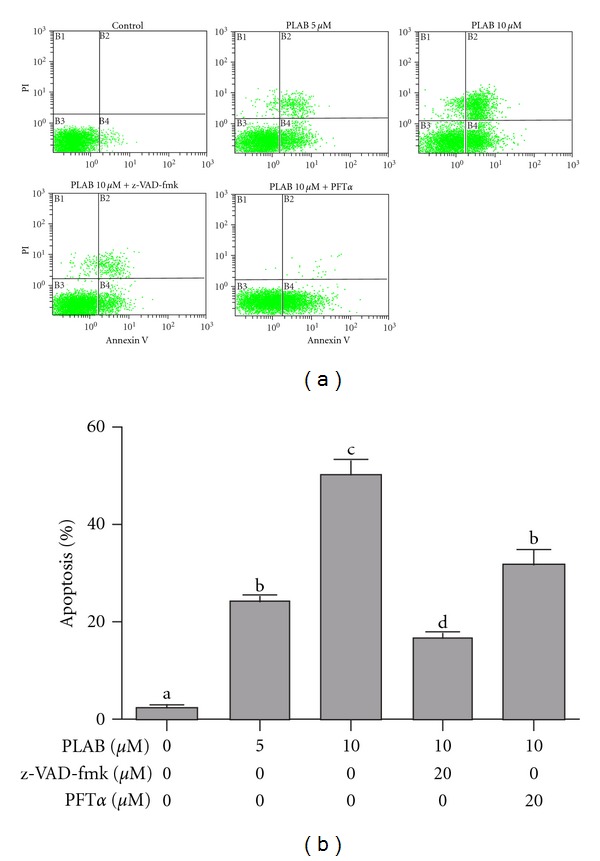
Flow cytometry analysis of apoptosis in U87 glioblastoma cells. (a) U87 cells were treated with indicated concentrations of PLAB in the presence or absence of z-VAD-fmk (20 *μ*M) or PFT*α* (20 *μ*M) for 24 h. After staining with Annexin V/PI, samples were analyzed by flow cytometry for apoptosis rates. (b) Data are expressed as Mean ± SEM (*n* = 3). Columns not sharing the same superscript letter differ significantly (*P* < 0.05).

DNA fragmentation and loss of plasma membrane asymmetry are the major features of apoptotic cell death. The effect of PLAB on cell death was analyzed by observing the nuclear morphological changes using Hoechst 33258 staining and fluorescent microscopy. As shown in Figure 3, PLAB induced obvious nuclear morphological changes including nuclear shrinkage and DNA fragmentation in U87 glioblastoma cells dose-dependently. Induction of apoptosis was further confirmed by Annexin V-FITC and PI staining. Treatment of cells with 5 and 10 *μ*M PLAB significantly increased apoptosis rate (24.43 ± 1.50 and 50.12 ± 3.42 versus 2.52 ± 0.49 in control group, *P* < 0.05) (Figure 4). Pretreatment with general caspase inhibitor (z-VAD-fmk) significantly reduced the apoptosis rate from 50.12 ± 3.42 to 16.92 ± 1.30 (*P* < 0.01) indicating that PLAB proceeds apoptosis in U87 glioblastoma cells mainly through caspase activation.Apart from caspase inhibitor, PFT*α*, a p53 inhibitor, also reduced the apoptosis rate from 50.12 ± 3.42 to 33.42 ± 2.85 indicating the involvement of p53 in PLAB-induced apoptosis in U87 glioblastoma cells (Figure 4).

### 4.3. PLAB Induces G2/M Phase Arrest in U87 Glioblastoma Cells

**Figure 5:**
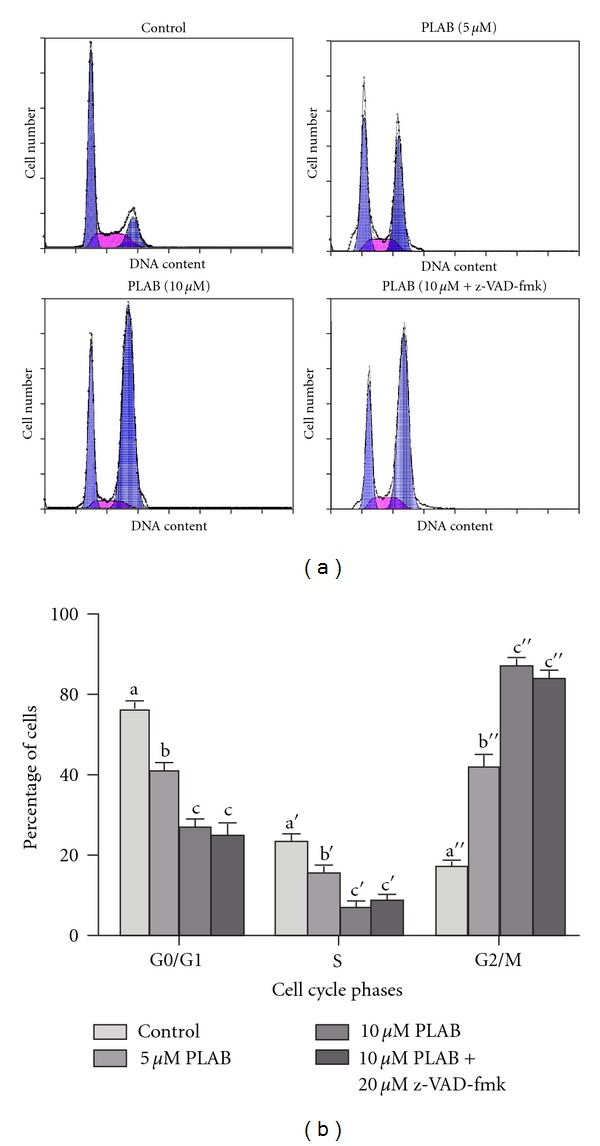
Flow cytometry analysis of cell cycle in U87 glioblastoma cells. (a) U87 glioblastoma cells were treated with DMSO or PLAB (5 and 10 *μ*M) in the presence or absence of z-VAD-fmk (20 *μ*M) for 24 h. Cells were collected, washed with PBS, and stained with a solution containing 50 *μ*g/mL PI and 100 *μ*g/mL RNase A for 30 min in the dark, at room temperature. Finally, samples were analyzed by flow cytometry for cell cycle phase distribution. (b) Data are expressed as Mean ± SEM (*n* = 3). Columns not sharing the same superscript letter differ significantly (*P* < 0.05).

The effect of PLAB on cell cycle profile was analyzed by PI staining and flow cytometry analysis. Treatment of PLAB at 5 and 10 *μ*M showed a dose-dependent increase in G2/M phase from 17.63 ± 2.06 to 42.25 ± 2.95 and 67.32 ± 1.83 respectively with a corresponding decrease in G0/G1 and S phase as shown in Figure 5.

### 4.4. PLAB Induces Apoptosis-Independent Cell Cycle Arrest in U87 Glioblastoma Cells

To further establish a link between apoptosis and cell cycle arrest, we performed apoptosis and cell cycle analysis using a general caspase inhibitor (z-VAD-fmk). As shown in Figure 4, caspase inhibitor significantly inhibited apoptosis rate but did not prevent mitotic arrest (Figure 5). The data suggest that cell cycle arrest by PLAB in U87 glioblastoma cells is an apoptosis-independent and early event in cell death mediated by PLAB.

### 4.5. PLAB Induces the Arrest of Mitotic Phase

Flow cytometry analysis of cell cycle distribution cannot differentiate G2 cells from mitotic cells as both cells in the G2 or mitotic phase possess 4N DNA contents. One previous study by Meng and Jiang [9] showed that PLAB induced G2 phase arrest in SK-28 melanoma cells via activation of ATM signalling pathway. Some other studies have shown that PLAB induces mitotic arrest by inhibiting tubulin polymerization [10, 14, 15]. 

**Figure 6:**
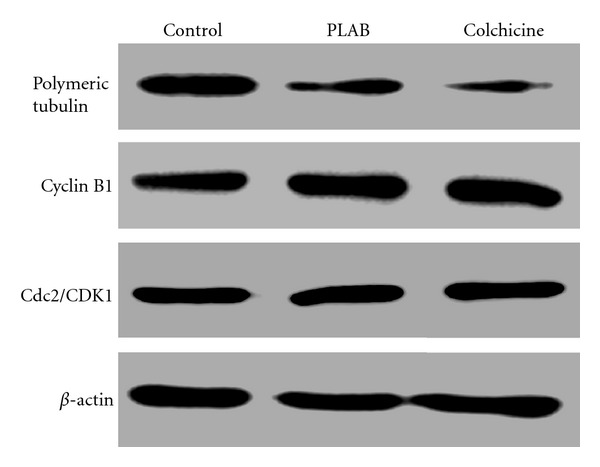
Effect of PLAB and colchicine (Reference drug) on G2/M phase cell cycle regulators in U87 glioblastoma cells. U87 glioblastoma cells were treated with 10 *μ*M PLAB and 3 *μ*M colchicine for 24 h. Total cell lysates and polymeric tubulin were extracted as described in Materials and Methods section. The expressions of polymeric tubulin, cyclin B1, and CDK1/Cdc2 were analyzed by Western blot analysis.

To investigate whether the inhibition of tubulin polymerization is involved in PLAB-induced G2/M phase arrest, we extracted polymeric tubulin from control and PLAB-treated U87 glioblastoma cells. The expression of polymeric tubulin was observed by Western blots. The results showed that PLAB downregulated polymeric tubulin in U87 glioblastoma cells (Figure 6). Colchicine, an inhibitor of tubulin polymerization was used as a positive control in this study. Colchicine demonstrated a similar inhibitory effect on tubulin polymerization in U87 glioblastoma cells (Figure 6). Furthermore, the expressions of proteins involved in G2/M phase arrest were examined by Western blot analysis. It is well documented that transition from G2 to M phase is triggered by the activation of the cyclin B1/Cdk1 complex. Cells with a suppressed cyclin B1/Cdk1 activity are arrested at G2 phase, whereas cells with an elevated cyclin B1/Cdk1 activity are favored to enter mitosis [18]. Therefore, U87 cells were treated with PLAB (10 *μ*M) and colchicine (3 *μ*M) and then harvested for Western blot analysis of cyclin B1 and Cdk1 expression levels. The data demonstrated that treatment of cells with PLAB or colchicine increased the expression of cyclin B1, whereas no change in the expression of Cdk1 (Cdc2) was observed (Figure 6). Taken together, the data suggested that PLAB induced cell cycle arrest in U87 glioblastoma cells at M phase but not at G2 phase.

### 4.6. PLAB Increases the Expression of p53 and Bax

**Figure 7:**
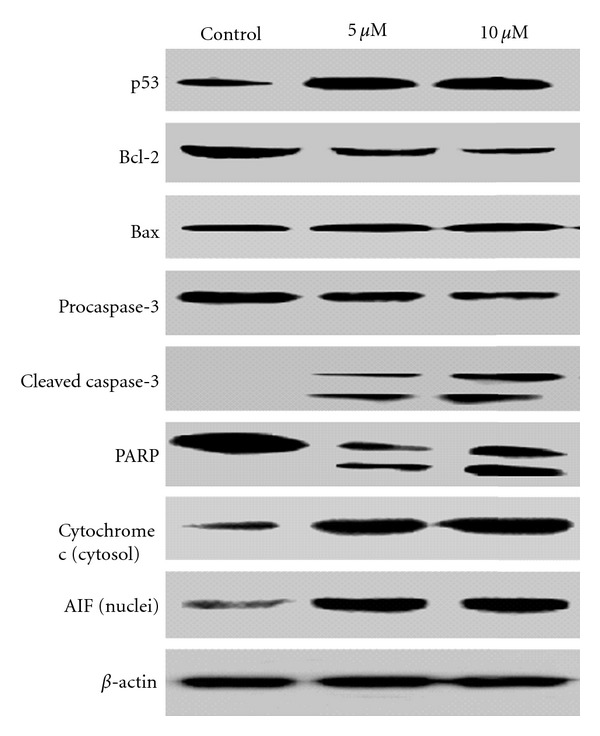
Effect of PLAB on apoptosis regulators in U87 glioblastoma cells. U87 glioblastoma cells were treated with indicated concentrations of PLAB for 24 h. Following treatment, cells were harvested and total, cytosolic and nuclear proteins were extracted as described in Materials and Methods section. The expression of p53, Bcl-2, Bax, Caspase-3, PARP, cytochrome c, and AIF were analyzed by Western blot analysis.

p53 is one of the most powerful tumor suppressor genes in human cancers. Since U87 glioblastoma cells express wild-type p53 and PFT*α*, a p53 inhibitor, reduced the apoptotic effect of PLAB, we wished to observe the expression of p53 in PLAB-treated U87 cells using Western blot. We found that PLAB markedly increased the expression of p53 in U87 cells in a dose-dependent manner (Figure 7). Since Bax is one of the crucial downstream mediators of p53 signalling, we observed the possible changes in the expression of Bax. An increased expression of Bax was found in PLAB-treated U87 cells. Apart from the induction of Bax, p53 activation has been shown to inhibit the expression of antiapoptotic protein Bcl-2 and our Western blot analysis revealed the same results (Figure 7).

### 4.7. PLAB Induces Cytochrome c Release and Caspase-3 Activation

To further define the apoptosis pathway, we measured the expression of cytochrome c and caspase-3 in U87 glioblastoma cells. The data showed that PLAB increased the expression of cytochrome c in cytosol and cleaved the caspase-3 into 17 kDa and 12 kDa proteins (Figure 7). To further confirm the involvement of caspase-3 in PLAB-induced apoptosis in U87 glioblastoma cells, we observed the expression of caspase-3 substrate, PARP using Western blot. Figure 7 shows the cleavage of PARP into 85 kDa protein. These findings clearly indicate that PLAB induces caspase-3-dependent apoptosis in U87 glioblastoma cells.

### 4.8. PLAB Partially Induces Caspase-Independent Apoptosis

As shown in Figure 4, the general caspase inhibitor, z-VAD-fmk did not inhibit the apoptotic effect of PLAB completely. This indicates that some caspase-independent apoptotic pathway is also involved. Apoptosis inducing factor (AIF) has been reported to induce caspase-independent apoptosis by directly inducing DNA fragmentation. We wished to check whether AIF is involved in PLAB-induced caspase-independent apoptosis in U87 cells. We examined the effect of PLAB on AIF nuclear translocation using Western blot. As shown in Figure 7, PLAB treatment increased the expression of AIF in nucleus dose-dependently. 

### 4.9. Determination of Organ Toxicity

**Figure 8:**
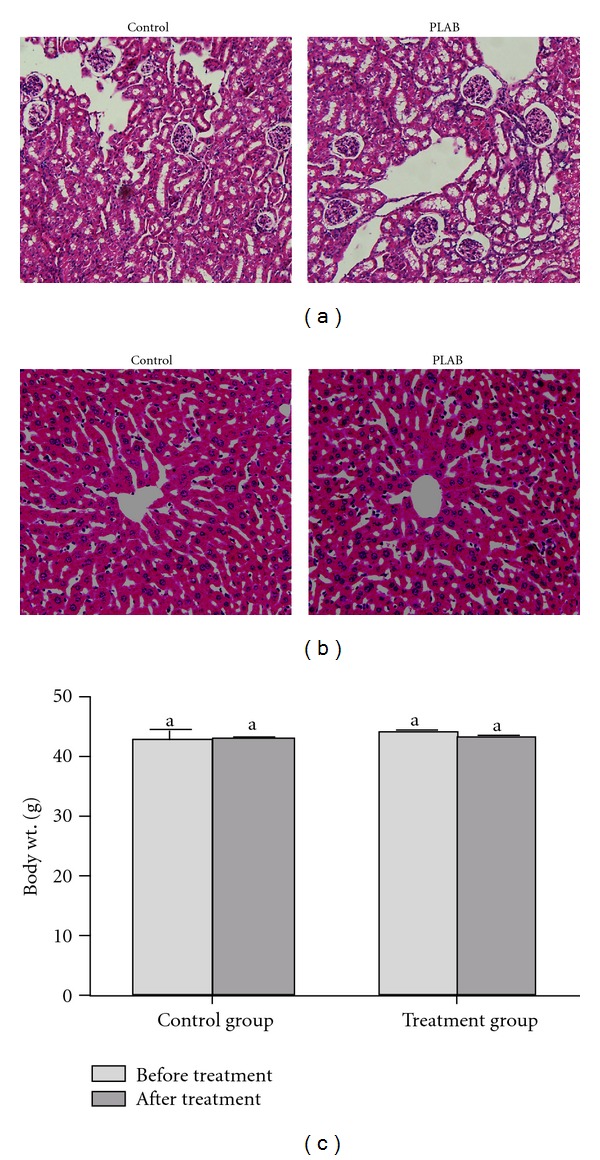
Effect of PLAB on liver and kidneys. Kunming mice were administered with vehicle or PLAB at a dose of 25 mg/kg body weight for 14 days. The liver and kidneys from control and PLAB-treated mice were excised and processed for hematoxylin and eosin staining followed established procedures. (a) kidney section, scale bar = 100 *μ*m; (b) liver sections, scale bar = 100 *μ*m. (c) Statistical analysis of body weights of experimental mice. Data are expressed as Mean ± SEM (*n* = 5). Columns not sharing the same superscript letter differ significantly (*P* < 0.05).

Hepatotoxicity and nephrotoxicity are the major side effects of cancer chemotherapeutic agents. Therefore, we investigated the effect of PLAB on liver and kidneys using Kunming mice. The cytotoxic effect of PLAB was assessed by measuring the changes in body weight, blood biochemistry and histopathology of liver and kidneys in comparison with control group. No obvious change in body weight of mice in treatment group has been observed when compared to control group. The histopathological changes in liver and kidneys were assessed using hematoxylin and eosin staining and correlated with liver and renal function biomarkers. No obvious morphological changes were observed in liver and kidneys structures of treatment group compared to control group (Figures 8(a) and 8(b)). These results were further confirmed by measuring the changes in liver function biomarkers (AST, ALT, and TBIL) and renal function biomarkers (BUN and Cr) in the serum of control and treatment groups. As shown in Table 1, there was a slight increase in serum ALT, AST and TBIL level of treatment group but this increase was not significantly different (*P* < 0.05) from control group. Similarly the changes in renal function biomarkers (Cr and BUN) were not significantly different (*P* < 0.05) in the serum of control and treatment groups. The concentration of Cr slightly increased whereas, concentration of BUN slightly decreased in treatment group (Table 1).

## 5. Discussion

An ideal cancer chemotherapeutic agent must not only kill the cancer cells but must in addition exhibit a high degree of selective toxicity between cancer cells and normal cells. Hepatotoxicity and nephrotoxicity are the major side effects of cancer chemotherapeutic drugs [19, 20]. An increasing number of studies in the past decade have shown that PLAB has a broad spectrum of cytotoxicity towards various human cancer cell lines of different origins [14]. In the present study, we investigated the inhibitory effect of PLAB on proliferation of U87 glioblastoma cells *in vitro* and simultaneously examined the toxic effect of this compound on liver and kidneys in animal mouse model. PLAB markedly inhibited the growth of U87 glioblastoma cells at low doses, however it did not display significant toxic effect on mouse liver and kidneys. 

Cell cycle arrest and apoptosis are the two main causes of growth inhibition [21]. Many anticancer agents exhibit their activity by inhibiting cell cycle progression at a particular checkpoint such as G0/G1, S, or G2/M and thereby induce apoptosis [22–25]. PLAB significantly arrested the cell cycle at G2/M phase in U87 glioblastoma cells in a dose-dependent manner. This result is consistent with previous studies that PLAB induced G2/M phase arrest in several types of human cancer cell lines [8–10, 14, 15]. Many anticancer drugs arrest the cell cycle at G2/M checkpoint either by damaging DNA or by disrupting mitotic spindles [23, 26, 27]. To gain further insight into molecular mechanism underlying PLAB-induced G2/M arrest in U87 glioblastoma cells, we observed the expression of polymeric tubulin in cells of control and treatment groups using Western blot analysis. The data showed that PLAB dramatically inhibited tubulin polymerization which indicated that PLAB arrested the cell cycle at mitotic phase but not at G2 phase. The results concur with those of Wong et al. [14] and Ma et al., [10] who demonstrated a similar effect of PLAB on other cancer cell lines. To provide further experimental evidence in support of the above notion that PLAB induced the cell cycle arrest at mitotic phase, we observed the expression of cyclin B1 and Cdk1 which are involved in progression of cell cycle from G2 to M phase. Cells with a suppressed cyclin B1/Cdk1 activity are arrested at G2 phase, whereas cells with an elevated cyclin B1/Cdk1 activity are favored to enter mitosis [18]. Our data demonstrated that PLAB increased the expression of cyclin B1 whereas no change in the expression of Cdk1 was observed. Colchicine, a known inhibitor of tubulin polymerization demonstrated a similar effect on the expression of cyclin B1 and Cdk1 in U87 cells. These results clearly demonstrate that PLAB induces mitotic arrest in U87 cells. 

One previous study by Wong et al. [14] showed that PLAB significantly inhibited the growth of tumor in nude mice at a dose of 15 mg/kg and 25 mg/kg without any sign of toxicity or body weight loss. However, they did not perform any *in vivo* study to examine the toxic effect of PLAB on normal body organs. In the present study, we examined the toxic effect of PLAB *in vivo* using Kunming mice. The data demonstrated that PLAB did not cause any detectable toxic effect in liver and kidneys at a dose of 25 mg/kg. Because cancer cells divide much more rapidly than normal cells, cancer cells are more susceptible to being poisoned by microtubule inhibitors than normal cells. The selective toxicity of PLAB between normal cells and cancer cells might be due to much more rapid division of cancer cells than normal cells. However, a detailed study for the molecular mechanism of selective cytotoxicity of PLAB still needs to be performed.

p53, a tumor suppressor protein, plays a key part in the regulation of cell cycle and cell death. p53 protein is also involved in cell differentiation, DNA repair, senescence, and angiogenesis. p53 has been shown to participate in both G0/G1 and G2/M checkpoints. p53 can also be activated in response to mitotic spindle damage [28]. In present study, an increased expression of p53 has been observed in U87 cells after treatment with PLAB. The activation of p53 in response to PLAB treatment is in agreement with previous studies [9, 11].

Once activated, p53 can induce the expression of several genes involved in apoptosis. In the present study, pretreatment of U87 glioblastoma cells with PFT*α*, (p53 inhibitor) attenuated the PLAB-mediated apoptosis significantly indicating that p53 upregulation is associated with induction of apoptosis. p53 has been reported to activate proapoptotic protein Bax and suppress antiapoptotic protein Bcl-2 [29–31]. Because proapoptotic stimuli induced by mitotic spindle damage involved in mitochondrial pathway [28], we wished to observe the expression of proteins involved in mitochondrial pathway using Western blot analysis. The data demonstrated that the expression of Bax gradually increased while the expression of Bcl-2 remarkably decreased with the release of cytochrome c from mitochondria to cytosol. These results are in line with previous reports that PLAB increases the expression of Bax and decreases the expression of Bcl-2 in Hela cells [12].

Once released, cytochrome c binds and activates caspase-9 which then leads to the activation of other downstream caspases and ultimately caspase-3 [32]. Activated caspases play an important role in apoptosis and cleave the PARP, a DNA repair enzyme. Activation of caspases and cleavage of PARP by caspases especially caspase-3 are the hallmarks of apoptosis [12]. Our data clearly demonstrate the cleavage of caspase-3 into 17 kDa and 12 kDa fragments and cleavage of PARP into 85 kDa fragment. These results clearly indicate that the intrinsic mitochondrial-mediated caspase activation pathway is involved in PLAB-mediated apoptosis in U87 glioblastoma cells. Our results are also supported by previous study that PLAB induced caspase-dependent apoptosis in Hela cells [12]. It is reported that the cell death induced by mitotic spindle damage is found to be both caspase-dependent and caspase-independent, because it can not be blocked completely by caspase inhibitor [2]. Our results confirm such a phenomenon clearly. In addition, PLAB have been shown to induce apoptosis and DNA fragmentation in MCF-7 cells that lack functional caspase-3 [8]. It has been documented that in addition to cytochrome c, mitochondria can also release the factors involved in caspase-independent cell death. Apoptosis inducing factor (AIF) is one of the major factors released from mitochondria and is believed to play a key role in the regulation of caspase-independent cell death [33] by binding to DNA, stimulating DNAse activity, and triggering chromatin condensation and DNA fragmentation [34]. In the present study, PLAB induced DNA fragmentation in U87 glioblastoma cells and z-VAD-fmk, a pharmacological broad-spectrum caspase inhibitor did not protect the cells from apoptotic cell death completely. These findings suggest the involvement of some other factors such as AIF, in caspase-independent cell death and our Western blot analysis clearly indicates the release of AIF from mitochondria and its translocation into nucleus in U87 glioblastoma cells after exposure to PLAB.

In conclusion, our data showed that PLAB induced mitotic arrest in U87 glioblastoma cells and consequently induced caspase-dependent apoptosis via up-regulation of p53 and Bax, down-regulation of Bcl-2 with release of cytochrome c and cleavage of caspase-3 and PARP and caspase-independent apoptosis through AIF. Moreover, PLAB did not cause significant toxicity in mouse liver and kidneys at a dose of 25 mg/kg. Therefore, PLAB may become a potential lead compound for future development of antiglioma therapy.

## Figures and Tables

**Table 1:** Serum Biomarker concentration for each group of experimental mice.

Groups	Dose (mg/kg)	ALT (U/L)	AST (U/L)	TBIL (*μ*mol/L)	Cr (*μ*mol/L)	BUN (mmol/L)
Control group	0	47.30 ± 3.70	79 ± 5.50	1.0 ± 0.12	27.4 ± 2.17	10.94 ± 3.01
Treatment group	25	55.45 ± 4.35 n.s.	87.66 ± 4.17 n.s.	1.2 ± 0.26 n.s.	36.5 ± 1.99 n.s.	9.45 ± 2.53 n.s.

Data are expressed as Mean ± SEM; n.s: not significant (*P* < 0.05).
