# Expression of Concern on “Pseudolaric Acid B Induces Caspase-Dependent and Caspase-Independent Apoptosis in U87 Glioblastoma Cells”

**DOI:** 10.1155/2019/4959838

**Published:** 2019-08-18

**Authors:** Evidence-Based Complementary and Alternative Medicine

*Evidence-Based Complementary and Alternative Medicine* would like to express concern with the article titled “Pseudolaric Acid B Induces Caspase-Dependent and Caspase-Independent Apoptosis in U87 Glioblastoma Cells,” published in March 2012 [1], due to a concern about cell line misidentification.

We became aware that the U87 cell line used in the article may be misidentified [2] and asked the authors to authenticate the cell line using Short Tandem Repeat (STR) profiling. They said they obtained the U87 cell line they had used from their former laboratory and prepared an STR analysis. However, the Editorial Board found that the STR analysis showed the cell line was likely not U87 or its derivatives because the ATCC matching score to U87 is only 73%; scores of 79–56% indicate a need for additional analysis [3]. There is a higher match at 79% to CCD-1068Sk, a female breast skin fibroblast cell line. The standard U87 cell line is XY, while their cell line is XX, which is concerning unless the authors can provide a karyotype analysis demonstrating that there was Y chromosome loss in their cell line.

More analyses are necessary for the confirmation of the actual cell line the authors used in this study and this notice may be updated or replaced based on the outcome of the analyses.

## References

[1] Khan M., Zheng B., Yi F. (2012). Pseudolaric Acid B Induces caspase-dependent and caspase-independent Apoptosis in U87 glioblastoma cells. *Evidence-Based Complementary and Alternative Medicine*.

[2] Allen M., Bjerke M., Edlund H., Nelander S., Westermark B. (2016). Origin of the U87MG glioma cell line: good news and bad news. *Science Translational Medicine*.

[3] ATCC, “Understanding the matching algorithm,” https://www.lgcstandards-atcc.org/understanding_the_matching_algorithm.aspx

