# Corrigendum to “Pseudolaric Acid B Induces Caspase-Dependent and Caspase-Independent Apoptosis in U87 Glioblastoma Cells”

**DOI:** 10.1155/2020/7939705

**Published:** 2020-10-08

**Authors:** Muhammad Khan, Bin Zheng, Fei Yi, Azhar Rasul, Zhuyi Gu, Ting Li, Hongwen Gao, Javed Iqbal Qazi, Hong Yang, Tonghui Ma

**Affiliations:** ^1^Central Research Laboratory, Jilin University Bethune Second Hospital, Changchun 130041, China; ^2^College of Life Sciences, Liaoning Normal University, Dalian 116029, China; ^3^Department of Zoology, University of the Punjab, Quaid-e-Azam Campus, Lahore, Pakistan

In the article titled “Pseudolaric Acid B Induces Caspase-Dependent and Caspase-Independent Apoptosis in U87 Glioblastoma Cells” [1], the identity of the cell line has been clarified following the publication of an Expression of Concern [2].

The laboratory director at the Jilin University Bethune Second Hospital obtained the original frozen cell line and sent the cells to a company for STR analysis (see the Supplementary Materials). The board is satisfied that the cell line tested is the glioblastoma cell line U87 or its derivative.

Supplementary Materials

Link to archivetempU87细胞STR鉴定实验报告 英文版.pdf: https://hindawihelp.freshdesk.com/helpdesk/attachments/48027005360?download=true.
